# Supplementary material for: Radiodynamic Therapy for High-Grade Glioma in Normoxic and Hypoxic Environments for High-Grade Glioma
Source: Cancers (Basel). 2025 Dec 8;17(24):3927. doi: 10.3390/cancers17243927 (PMC12730920; doi:10.3390/cancers17243927)
Supplement: Supplementary file 1 [file cancers-17-03927-s001.zip › cancers-4027297-supplementary.pdf]

Radiodynamic Therapy for High-Grade Glioma in Normoxic and Hypoxic Environments for High-Grade Glioma

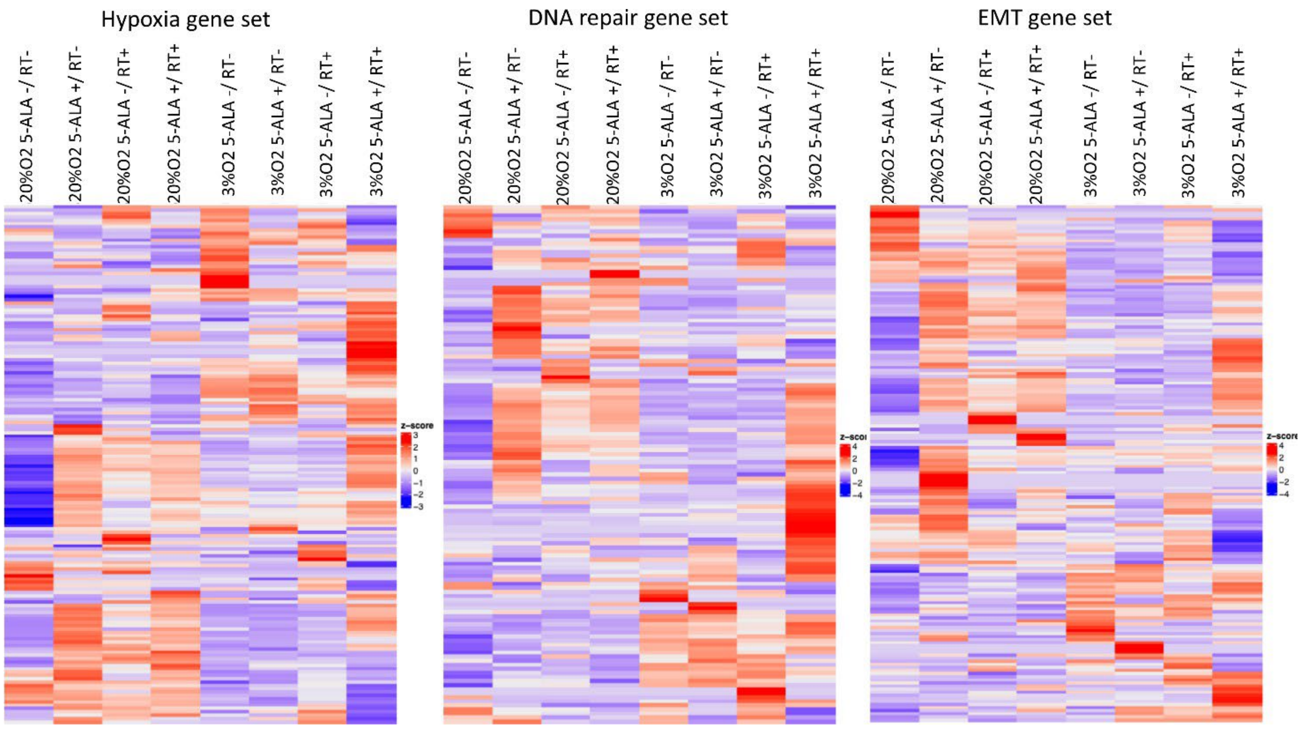

Supplementary Figure S1. RNA-seq heatmap using hypoxia, DNA repair, and EMT gene sets

Heatmaps showing RNA-seq expression profiles for hypoxia-related, DNA repair, and epithelial–mesenchymal transition (EMT) gene sets in U87 cells under different treatment and oxygen conditions. RNA was extracted from U87 cells that were untreated (5-ALA-/RT-), treated with 5-ALA (1000 μM) only (5-ALA+/RT-), irradiated with 6 Gy X-rays only (5-ALA-/RT+), or treated with both 5-ALA and X-ray irradiation (5-ALA+/RT+). The cells were cultured for 7 days in either a 20% O_2 environment (normoxia) or a 3% O_2 environment (hypoxia). Gene expression levels were normalized using the transcripts per million (TPM) method and visualized in color-mapped heatmaps, where red indicates high expression levels, blue indicates low expression levels, and shades in between represent intermediate expression levels. The heatmaps illustrate the differential expression of genes related to hypoxia, DNA repair, and EMT across varying treatment and oxygenation conditions.
